# Analysis and interpretation of joint source separation and sound event detection in domestic environments

**DOI:** 10.1371/journal.pone.0303994

**Published:** 2024-07-05

**Authors:** Diego de Benito-Gorrón, Katerina Zmolikova, Doroteo T. Toledano

**Affiliations:** 1 AUDIAS Research Group, Escuela Politécnica Superior, Universidad Autónoma de Madrid, Madrid, Spain; 2 Faculty of IT, IT4I Centre of Excellence, Brno University of Technology, Brno, Czech Republic; Mae Fah Luang University, THAILAND

## Abstract

In recent years, the relation between Sound Event Detection (SED) and Source Separation (SSep) has received a growing interest, in particular, with the aim to enhance the performance of SED by leveraging the synergies between both tasks. In this paper, we present a detailed description of JSS (Joint Source Separation and Sound Event Detection), our joint-training scheme for SSep and SED, and we measure its performance in the DCASE Challenge for SED in domestic environments. Our experiments demonstrate that JSS can improve SED performance, in terms of Polyphonic Sound Detection Score (PSDS), even without additional training data. Additionally, we conduct a thorough analysis of JSS’s effectiveness across different event classes and in scenarios with severe event overlap, where it is expected to yield further improvements. Furthermore, we introduce an objective measure to assess the diversity of event predictions across the estimated sources, shedding light on how different training strategies impact the separation of sound events. Finally, we provide graphical examples of the Source Separation and Sound Event Detection steps, aiming to facilitate the interpretation of the JSS methods.

## Introduction

An important part of the information we obtain from our surroundings is carried by sound, helping us to understand where we are or what is happening around us. With this motivation, several research fields in audio signal processing aim to exploit the contents of sound signals to retrieve information about the environment. For instance, Sound Event Detection (SED) [1] answers the questions of which are the specific events that occur in an audio recording, and when do they begin and end. Other related tasks are Acoustic Scene Classification [2], which labels an audio according to the environment it has been captured in (e.g. house, park, train station), or Automated Audio Captioning [3], which aims to provide a text description of the recording.

With the objective of supporting the research in SED and other environmental sound analysis tasks, yearly challenges are hosted by the DCASE community (Detection and Classification of Acoustic Scenes and Events [4]), suggesting research questions and offering standard frameworks in which different approaches can be compared. Among the focuses of the SED task in recent DCASE challenges, the main one is the exploitation of unlabeled or partially-annotated data to train SED systems [5], whereas complementary lines of research have been proposed, such as the use of Source Separation (SSep) to aid Sound Event Detection [6].

The interest in leveraging data with different degrees of annotation is related to the high cost of data curation and annotation, in comparison to the wide availability of unlabeled data. An illustrative example is AudioSet [7], a large-scale acoustic event dataset containing more than 2 million 10-seconds audio clips, which has increased the viability of deep learning algorithms in SED. The audio clips in AudioSet are extracted from YouTube videos, and are provided with weak annotations (i.e. clip-level labels) or strong annotations [8] (i.e. timestamps of the onset and offset of each event), according to a comprehensive ontology that organizes and describes more than 500 categories of acoustic events. A similar distribution of data annotations is observed in DESED (Domestic Environment Sound Event Detection) [9, 10], the dataset employed in the DCASE challenge, which is composed of weakly-labeled and strongly-labeled audio clips, in addition to a majority of unlabeled examples.

With the aim of leveraging unlabeled examples and reducing the dependency on annotated data, semi-supervised and unsupervised learning methods have been developed, currently being evolving fields of research. Unsupervised learning is able to learn using only unlabeled data, whereas semi-supervised learning (SSL) leverages both labeled and unlabeled examples, reducing the amount of necessary annotations [11]. In the context of DCASE challenges, the most common SSL algorithm for SED is Mean Teacher, which considers a moving average version (teacher) of the original model (student), and then incorporates the consistency between student and teacher predictions as part of the loss function used to train the student model [12].

Another research interest in DCASE is the use of Source Separation (SSep) to enhance Sound Event Detection. SSep aims to automatically decompose audio mixtures into their underlying components, considering that each component has been produced by a different acoustic source. For example, SSep can isolate the speech signal in a recording that contains speech and background noise, or separate the different instruments in a music mixture. Therefore, the application of SSep to SED serves the purpose of decomposing audio event mixtures into several audio channels, each of them containing lower levels of noise or less overlap of target events, being more adequate inputs to the SED system.

Following this approach, the first method proposed by DCASE involved convolutional masking networks trained for SSep as a pre-processing step for SED [6]. A late integration of SSep and SED, combining the SED outputs for the mixture and separated sound sources, was observed to be more beneficial than an early integration (concatenating the separated sources and the mixture as a multi-channel input to the SED model), or a middle integration (concatenating intermediate representations). However, the method provided limited improvements, which was explained by a mismatch between the training data of the SSep model (artificial sound mixtures) and the test data of SED (web audio). For the 2021 challenge, DCASE proposed a baseline system that also used a late fusion of pre-trained SSep and SED, but using a Source Separation model trained over web audio by means of Mixture Invariant Training (MixIT) [13], an unsupervised method for training SSep neural networks.

More recent works have explored the combination of Source Separation and Sound Event Detection or classification. Some of them, in a similar way to DCASE approaches, use Source Separation to enhance the performance of Sound Event Detection systems. For instance, training a Source Separation network from scratch using a task-aware objective [14], or encouraging a SED system to separate sources in its intermediate representations [15]. In contrast, other works aim for the opposite direction: employing the information provided by sound classifiers to aid the separation of mixtures into semantically different sources [16–18].

Considering that Source Separation can improve Sound Event Detection, and that the information provided by a SED system might be helpful for Source Separation, a training setting which encourages a bidirectional flow of information between SSep and SED seems to be an interesting approach. In previous work [19], we have proposed Joint Sound Event Detection + Source Separation (JSS), a joint model in which a Source Separation block is connected to a Sound Event Detection block. After pre-training each block independently for its correspondent task, the whole model is trained end-to-end using Sound Event Detection objectives, either together (Joint Training) or in a separate stage for each block (Two-stage Training). Moreover, the development of JSS implied an exploration of the model selection strategy employed for the semi-supervised Mean Teacher models, which is especially relevant for iterative training processes. We found that our methods were able to improve SED performance in the context of DCASE Challenge Task 4, especially when the Source Separation block was pre-trained using in-domain data.

Building upon previous research, this paper introduces the following main contributions: (1) We offer a comprehensive definition of the JSS method and its different variants (Joint Training and Two-stage Training), including as well the proposed model selection criterion for Mean Teacher, which is proven to enhance the results. (2) We provide results and analysis of JSS over two additional datasets: DESED Public evaluation, and Public overlap (an overlapped version of the aforementioned dataset, introduced in our previous work [20]). (3) We analyze and discuss several aspects of the JSS method which were not covered by previous works, including its performance for specific sound event categories, or the role of Source Separation in the system, which we measure by means of the similarity of SED predictions across estimated sources. (4) Finally, we offer graphical representations of the intermediate steps of the systems, which aid understanding of the interactions between Sound Event Detection and Source Separation and enhance the interpretability of the resulting systems.

## Sound Event Detection

**Fig 1:**
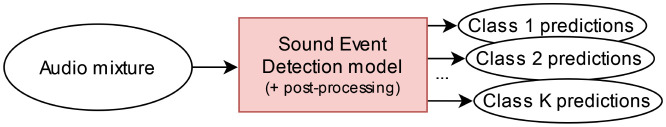
Block diagram of Sound Event Detection (SED). A Sound Event Detection system computes, for an input audio mixture, the temporal boundaries of a set of *K* event categories.

Sound Event Detection aims to obtain, for an input audio signal **x**, the time boundaries (*t*_on_, *t*_off_) for a closed set of *K* acoustic event classes (Fig 1). A common approach is to consider the problem as *K* binary classification tasks in time, so that a detection score sequence ${\hat{d}}_{k}\in {\left(0,1\right)}^{T}$ is estimated for each event category, with length *T*. This set of *K* score sequences forms a matrix $\hat{D}\in {\left(0,1\right)}^{K\times T}$.

A usual approach for neural network SED systems is to obtain $\hat{D}$ by means of a *K*-dimensional output layer with sigmoid activation. In such case, the score sequences $\hat{D}$ are a function of the input signal **x**, and the model parameters ***θ***_sed_ (Eq (1)).

$$\begin{array}{c} \hat{D}=f^{\text{(sed)}}\left(x;\theta_{\text{sed}}\right) \end{array}$$ (1)

Once $\hat{D}$ is computed, the values of the onset and offset times can be determined by defining a threshold *τ* ∈ (0, 1). Then, for each event category *k* ∈ [1, *K*], the onsets *t*_on,*k*_ are the times when the score goes above the threshold (${\hat{d}}_{k}\left(t_{\text{on},k}\right)\geq \tau ,{\hat{d}}_{k}\left(t_{\text{on},k}-1\right)<\tau$), and the offsets *t*_off,*k*_ correspond to the time frames when the score becomes lower than the threshold (${\hat{d}}_{k}\left(t_{\text{off},k}\right)<\tau ,{\hat{d}}_{k}\left(t_{\text{off},k}-1\right)\geq \tau$). As a post-processing step, a median filter is applied after thresholding, in order to avoid spurious onsets and offsets.

### Sound Event Detection in DCASE challenge

**Fig 2:**
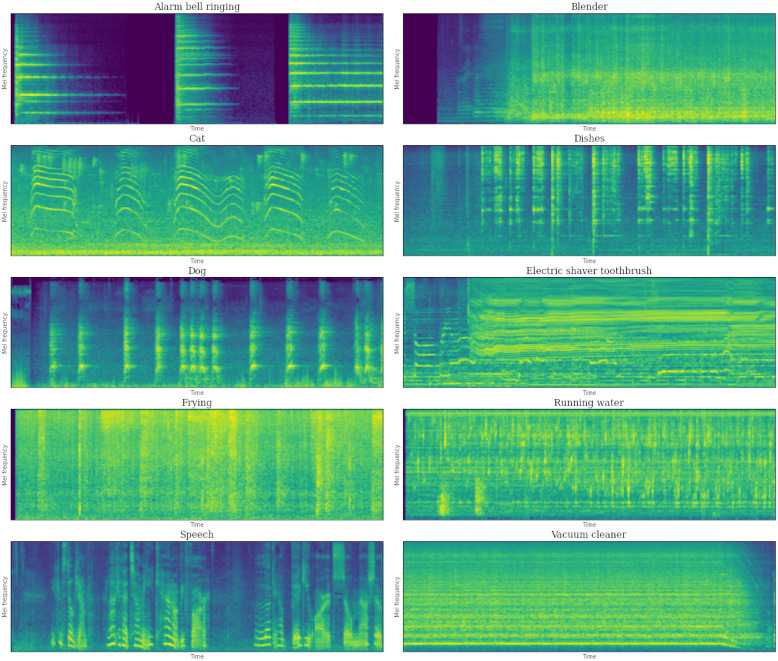
Sample mel-spectrogram representations of the 10 different target event categories considered in DCASE Challenge Task 4. The represented audio segments are extracted from the DCASE Public evaluation set, considering segments in which a unique target event is annotated.

The scope of Sound Event Detection in the DCASE Challenge Task 4 is focused on domestic environments, which are especially relevant for indoor applications such as home assistance or security. In this direction, a set of 10 event categories drawn from the AudioSet ontology is considered: *Alarm/bell/ringing*, *Blender*, *Cat*, *Dishes*, *Dog*, *Electric shaver/toothbrush*, *Frying*, *Running water*, *Speech*, and *Vacuum cleaner*. An example of a mel-spectrogram representation of each event category is provided in Fig 2.

Several research questions have been stated in the recent editions of DCASE Challenge Task 4, most of them regarding the use of different types of training data, such as a large amount of unlabeled audio clips from web videos or strongly-labeled synthetic recordings [21], in addition to a small set of weakly-labeled data. For this purpose, semi-supervised learning approaches were the main focus, with Mean Teacher [12] becoming a standard approach thanks to its simplicity and good results [22].

A SED Baseline system is provided each year by the challenge, aiming to establish a performance benchmark, and including some advances of the state of the art. The current baseline is a Convolutional-Recurrent Neural Network (CRNN) [23].

**Fig 3:**
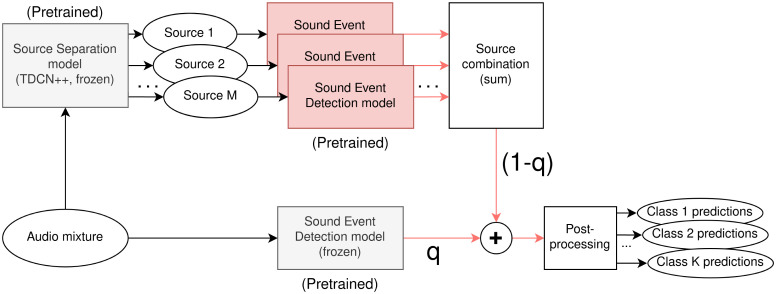
Block diagram of the DCASE 2021 Baseline system for Source Separation + Sound Event Detection (SSep-SED). The weight *q* is learnt during the training process. The parameters of the frozen blocks are not updated during the training process.

In 2020, the challenge proposed Source Separation for SED as an auxiliary task, called Sound Event Separation and Detection (SSep+SED). This task involved the use of SSep systems to separate overlapping sound events and extract foreground sound events from the background sound, and in it introduced an additional Baseline system, described in Fig 3. In order to train the SSep+SED Baseline system, the training data is first separated using a pre-trained Source Separation network. Then, the SED baseline system, already trained over the original mixtures, is fine-tuned to the separated data. In order to obtain the final score sequences, the outputs of the fine-tuned SED system over separated sources, ${\hat{D}}_{\text{sep}}$, are combined with the outputs of the pre-trained SED Baseline over the mixtures ${\hat{D}}_{\text{mix}}$, as described in Eq (2). The combination weight, *q*, is learnt during the fine-tuning process.

$$\begin{array}{c} {\hat{D}}_{\text{ssep+sed}}=q{\hat{D}}_{\text{mix}}+\left(1-q\right){\hat{D}}_{\text{sep}} \end{array}$$ (2)

In the DCASE SSep+SED Baseline, both the SSep network and the SED model that is applied over the mixtures are frozen, meaning that their parameters are not updated during the training process.

### Semi-supervised Sound Event Detection with Mean Teacher

The scarcity of annotations in Sound Event Detection training data can be solved by means of a semi-supervised learning algorithm. Particularly, Mean Teacher is the method proposed by the DCASE Baseline system.

Mean Teacher training considers two models, student and teacher, with identical structure. The weights of the student model, ***θ***^(s)^, are trained with back-propagation using a loss function *L*_sed_, whereas the weights of the teacher model, ***θ***^(t)^, are computed at each training step *n* as an exponential moving average (EMA) of ***θ***^(s)^ (Eq (3)).

$$\begin{array}{c} \theta_{n}^{\text{(t)}}=\alpha_{\text{ema}}\;\theta_{n-1}^{\text{(t)}}+\left(1-\alpha_{\text{ema}}\right)\;\theta_{n}^{\text{(s)}} \end{array}$$ (3)

The weight *α*_ema_ ∈ (0, 1) is a hyperparameter that determines the exponential decay, with higher values resulting in slower updates of the teacher model. Its optimal value can be obtained empirically.

In order to leverage the information provided by both labeled and unlabeled data, the loss function *L*_sed_ is divided into two components:

- A supervised loss (*L*_sup_, Eq (4)), which is implemented as a Binary Cross-Entropy between the student score sequences (${\hat{D}}^{\text{(s)}}$) and the ground truth annotations, **D**.
  $$\begin{array}{c} L_{\text{sup}}=\text{BCE}\left({\hat{D}}^{\text{(s)}},D\right) \end{array}$$ (4)
- A self-supervised consistency loss (*L*_self_, Eq (5)), computed as the Mean Squared Error between student and teacher predictions.
  $$\begin{array}{c} L_{\text{self}}=\text{MSE}\left({\hat{D}}^{\text{(s)}},{\hat{D}}^{\text{(t)}}\right) \end{array}$$ (5)

Whereas *L*_sup_ can only be computed for labeled examples, *L*_self_ does not require ground truth annotations. This allows the models to learn from all training examples.

The global loss function for Sound Event Detection, *L*_sed_, is computed as a weighted sum of both components (Eq (6)), and used to train the student model. The weight of the self-supervised loss (*α*_self_) regulates the contribution of the consistency measure.

$$\begin{array}{c} L_{\text{sed}}=L_{\text{sup}}+\alpha_{\text{self}}L_{\text{self}} \end{array}$$ (6)

Therefore, a feedback loop is created between student and teacher: the student model is trained to minimize a loss function that considers consistency with teacher predictions, while the teacher is computed as a smoothed (averaged) version of the student.

### Metrics and evaluation of Sound Event Detection systems

#### *F*_1_-score-based metrics

*F*_1_-score (Eq (7)) is a widely adopted metric to measure the performance of SED systems. It can be computed as the harmonic mean of Precision (P) and Recall (R), defined in Eqs (8) and (9) respectively. Thus, taking into account the definitions of Precision and Recall, *F*_1_-score ultimately depends on the number of True Positive (TP), False Negative (FN), and False Positive (FP) decisions of the system.

$$\begin{array}{c} F_{1}=2\;\frac{PR}{P+R} \end{array}$$ (7)

$$\begin{array}{c} P=\frac{\text{TP}}{\text{TP}+\text{FP}} \end{array}$$ (8)

$$\begin{array}{c} R=\frac{\text{TP}}{\text{TP}+\text{FN}} \end{array}$$ (9)

In DCASE Task 4, the main *F*_1_ score is event-based (or collar-based), meaning that the decisions of the system are measured for entire occurrences of an event (TP, FN) or a prediction (FP), considering a certain tolerance between the system predictions and the ground truth annotations.

#### Polyphonic Sound Detection Score

In recent editions of DCASE Task 4, Polyphonic Sound Detection Score (PSDS) [24] is proposed as a performance metric for SED. The aim of PSDS is to solve some limitations of *F*_1_-scoring, particularly the dependence on a single decision threshold, the lack of robustness to annotation subjectivity, and the agnosticism to cross-triggered detections.

To handle these problems, PSDS is defined as the area under a curve determined by the performance of the system at different thresholds, considering a True Positive Rate and a False Positive Rate. In contrast with event-based and segment-based *F*_1_, TPs and FPs follow an intersection criterion, more robust to variability of the ground truth labels. Finally, cross-triggers are defined in PSDS as FP decisions that coincide with a different target category, and are considered as a different kind of error.

An additional contribution of PSDS is the definition of several parameters that allow to adapt the metric to different applications or scenarios. The Detection Tolerance Criterion (DTC) and Ground Truth Intersection Criterion (GTC) define the amount of intersection between predictions and annotations that is necessary to consider a correct detection, and the Cross-Trigger Tolerance (cttc) establishes the threshold for cross-triggered decisions. The penalty introduced by cross-triggers (*α*_CT_) and a cost for instability between classes (*α*_ST_) can also be configured.

DCASE Task 4 proposes two different configurations for PSDS. The first scenario (PSDS1) encourages systems to make a finer temporal segmentation of the events, whereas the second scenario (PSDS2) is more focused on classification accuracy. Both of them are computed using 50 threshold values, linearly distributed from 0 to 1. The parameter settings for each scenario are described in Table 1.

**Table 1:** Parameter configuration for the PSDS scenarios. DTC = Detection Tolerance Criterion. GTC = Ground Truth intersection Criterion. *α*_ST_ = Cost of instability across classes. CTTC = Cross-Trigger Tolerance Criterion. *α*_CT_ = Cost of Cross Triggers. *e*_max_ = Maximum False Positive Rate.

Scenario	DTC	GTC	*α*_ST_	CTTC	*α*_CT_	*e*_max_
PSDS1	0.7	0.7	1.0	0.0	-	100
PSDS2	0.1	0.1	1.0	0.3	0.5	100

## Source separation

**Fig 4:**
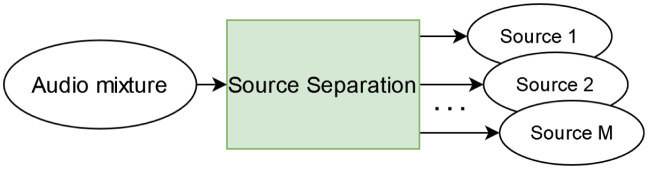
Block diagram of Source Separation (SSep). A Source Separation system decomposes the input audio mixture into a predefined number (*M*) of estimated sources.

Acoustic Source Separation (or Source Separation) can be stated as a regression task, in which an input sound mixture **x** is decomposed into *M* estimates, $\hat{S}=\langle {\hat{s}}_{m}\rangle ,1\leq m\leq M$, of its underlying source components (Fig 4). Additionally, a consistency constraint is usually applied, so that the sum of output sources is equivalent to the input mixture, $\sum_{m=1}^{M}{\hat{s}}_{m}=x$. Considering a separation model *f*^(sep)^, with parameters ***θ***_sep_, the estimate is obtained as shown in Eq (10).

$$\begin{array}{c} \hat{S}=f^{\text{(sep)}}\left(x;\theta_{\text{sep}}\right) \end{array}$$ (10)

Given that the goal is to reproduce the reference sources, SSep models can be trained employing a negative Signal-to-Noise Ratio (SNR) loss (Eq (11)), in which the target signals are the reference sources **s**, and the noise is the error $s-\hat{s}$. A small quantity *ϵ* is added to this term, to prevent the division by zero in the case that $\hat{s}=s$.

$$\begin{array}{c} L_{\text{sep}}\left(s,\hat{s}\right)=-10\;{\text{log}}_{10}(\frac{{\left||s|\right|}^{2}}{||s-\hat{s}{\left|\right|}^{2}+\epsilon }) \end{array}$$ (11)

Several tasks involve Acoustic Source Separation with different kinds of audio mixtures, or different constraints on the acoustic sources of interest. Some examples are music source separation [25], which aims to isolate the different instruments present in a music signal, speech separation [26], in which a mixture of various speakers is divided into individual speech signals for each speaker, or speech enhancement [27], consisting on improving the quality of a noisy speech signal by removing the non-speech content, thus separating the input mixture into an only-speech channel and a non-speech channel.

An additional application is Universal Source Separation, defined as the decomposition of any acoustic soundscape into sounds of arbitrary types [28]. In this scenario, some particularities have to be considered, for instance, the order of the output signals should not be relevant (permutation problem), and the number of sources is inherently unknown. Moreover, in order for a model to learn the separation of arbitrary classes, the training data should include a great diversity of sounds.

### Supervised Source Separation with Permutation Invariant Training

Permutation Invariant Training (PIT) was introduced as a solution to the permutation problem [29]. Considering a loss function $L_{\text{sep}}\left(S,\hat{S}\right)$, PIT compares all the possible permutations of the estimated sources $\hat{S}$ (using the permutation matrix **P**) with the targets **S**, and takes the minimum loss value as the result, thus making the training process independent of the order of the outputs. The PIT loss is thus defined in Eq (12).

$$\begin{array}{c} L_{\text{PIT}}\left(S,\hat{S}\right)=\underset{P}{\text{min}}\sum_{m=1}^{M}L_{\text{sep}}\left(s_{m},{\left[P\hat{S}\right]}_{m}\right) \end{array}$$ (12)

In order for a SSep model with a fixed number of outputs (*M*) to deal with a variable number of target sources during training, a new loss function is proposed in [30], dividing *L*_sep_ into two SNR-based terms: an active loss (*L*_a_) and an inactive loss (*L*_0_). When applying PIT, the active loss is derived from the negative SNR loss (Eq (11)), and computed with respect to the *M*_*a*_ active target sources (*M*_*a*_ ≤ *M*), as shown in Eq (13), whereas the inactive SNR loss is applied with respect to *M* − *M*_*a*_ null signals, aiming to minimize the separated source power of inactive channels (Eq (14)).

$$\begin{array}{c} L_{\text{a}}\left(s,\hat{s}\right)=10\;{\text{log}}_{10}(||s-\hat{s}{\left|\right|}^{2}+{\tau ||s||}^{2}), \end{array}$$ (13)

$$\begin{array}{c} L_{\text{0}}\left(x,\hat{s}\right)=10\;{\text{log}}_{10}\left(|\right|\hat{s}{\left|\right|}^{2}+{\tau ||x||}^{2}). \end{array}$$ (14)

The parameter *τ* in Eqs (13) and (14) is introduced as a soft threshold to determine the maximum SNR value, aiming to prevent large gradients from dominating the total loss.

Thus, the PIT loss with a variable number of target sources is defined in Eq (15).

$$\begin{array}{c} L_{\text{PIT}}\left(S,\hat{S}\right)=\underset{P}{\text{min}}\sum_{m=1}^{M_{a}}L_{a}\left(s_{m},{\left[P\hat{S}\right]}_{m}\right)\;+\;\sum_{m_{0}=M_{a}+1}^{M}L_{\text{0}}\left(x,{\left[P\hat{S}\right]}_{m_{0}}\right) \end{array}$$ (15)

Regarding the availability of training data for Universal Source Separation, the main limitation is the access to the reference sources of the mixtures, which generally are not possible to obtain. However, it is possible to create artificial training mixtures by overlapping several isolated sources, which can then be used as targets, as in the case of the FUSS dataset (Free Universal Source Separation) [30].

### Unsupervised Source Separation with Mixture Invariant Training

A different approach is to train Source Separation in an unsupervised fashion. In this way, reference sources are not necessary to train the models, allowing the use of non-artificial datasets. This is the motivation of Mixture Invariant Training (MixIT), an unsupervised learning algorithm for Source Separation based on the use of “mixtures of mixtures”. MixIT proposes to use the sum of two audio mixtures, **x**_1_ + **x**_2_, as input to the model, so that the *M* outputs of the model, $\hat{S}$, are expected to contain the underlying sources of **x**_1_ and **x**_2_ (Eq (16)).

$$\begin{array}{c} \hat{S}=f^{\text{(sep)}}\left(x_{1}+x_{2};\theta_{\text{sep}}\right) \end{array}$$ (16)

Given that reference sources for the mixtures are not available, MixIT computes all possible assignations of each output to either **x**_1_ or **x**_2_, by means of a binary assignation matrix **A**, with size (2, *M*), in which each column sums up to one. The MixIT loss is then computed in Eq (17) as the minimal loss considering every possible assignation between outputs and inputs.

$$\begin{array}{c} L_{\text{MixIT}}\left(x_{1},x_{2},\hat{S}\right)=\underset{A}{\text{min}}\sum_{i=1}^{2}L_{\text{sep}}\left(x_{i},{\left[A\hat{S}\right]}_{i}\right) \end{array}$$ (17)

Whereas this method overcomes the need of target sources to train a separation model, which is the main limitation of PIT, it raises some problems, the main one being a tendency for over-separation, in which a single source is decomposed into several output signals, generally leading to more active output sources than necessary. This occurs because the MixIT loss is blind to the content of individual estimated sources ${\hat{s}}_{m}$, as long as a good reconstruction of the input mixtures is possible. More recent additions to MixIT have dealt with this problem by including penalties for over-separation, such as sparsity loss or covariance loss [18].

### Source Separation with mask estimation neural networks

The decomposition of a sound mixture **x** into several components can be approached as a mask estimation problem in the time-frequency domain, dividing the task into three stages: the transformation of the audio signal **x** into a time-frequency representation with an encoder function E, the computation of the mask for each source, **W**_*m*_, and the reconstruction of the estimated sources $\hat{S}$ in the time domain with a decoder function D. The encoder and decoder functions can either be pre-defined transformations (e.g. Short-Time Fourier Transform) or learnt from data.

The masks are weights that control the contribution of the mixture to each estimated source, so that each source ${\hat{s}}_{m}$ can be obtained by performing an element-wise multiplication (⊙) between the encoded input (E(x)) and the corresponding mask **W**_*m*_, and then decoding the result with the decoder function D, as described in Eq (18).

$$\begin{array}{c} {\hat{s}}_{m}=D\left(E\left(x\right)⊙W_{m}\right) \end{array}$$ (18)

An example of mask estimation neural network for Source Separation is ConvTas-Net [31]. In particular, its architecture is formed by a fully-convolutional separation module, with several repeats of convolutional blocks with increasing dilation factors. The masks are estimated with a pointwise convolution, and the encoder and decoder functions are learnt during training.

### Metrics and evaluation of source separation systems

In order to evaluate the performance of Source Separation models, a standard approach is to measure the mean SNR improvement (SNRi, Eq (19)) of the estimated sources ($\hat{s}$) to their corresponding reference signals (**s**), with respect to the SNR obtained when using the mixture (**x**) as estimation. In order to prevent divisions by zero and ensure numerical stability, an infinitesimally small positive quantity, *ϵ*, is added to the denominators in Eq (19).

$$\begin{array}{c} \text{SNR}i\left(\hat{s},s,x\right)=10\;{\text{log}}_{10}\frac{{\left||s|\right|}^{2}}{||s-\hat{s}{\left|\right|}^{2}+\epsilon }-10\;{\text{log}}_{10}\frac{{\left||s|\right|}^{2}}{||s-{x||}^{2}+\epsilon } \end{array}$$ (19)

## Proposed methods

### Joint Source Separation + Sound Event Detection

**Fig 5:**
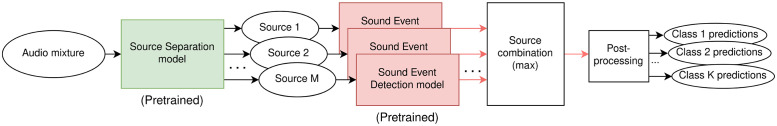
Block diagram of Joint Source Separation + Sound Event Detection (JSS).

Considering the potential mutual benefits of the tasks of Source Separation and Sound Event Detection, we aim to complement both in a single system, called Joint SSep + SED (JSS). Such a system receives an audio mixture as input and computes the temporal boundaries of sound events, in the same manner as a traditional SED system, but with the difference that the predictions are obtained from automatically separated sources of the mixture, which are computed during the same inference process (Fig 5).

The proposed JSS systems consist, then, of a Source Separation block (*f*^(sep)^) that divides the input mixture into *M* sources (Eq (20)), and a Sound Event Detection block (*f*^(sed)^) that obtains event score sequences (${\hat{D}}_{m}$) for each of the estimated sources, considering *K* event classes (Eq (21)).

$$\begin{array}{c} f^{\text{(sep)}}\left(x;\theta_{\text{sep}}\right)=\hat{S}={\left\langle {\hat{s}}_{m}\right\rangle }_{m=1}^{M}, \end{array}$$ (20)

$$\begin{array}{c} f^{\text{(sed)}}\left({\hat{s}}_{m};\theta_{\text{sed}}\right)={\hat{D}}_{m}={\left\langle {\hat{d}}_{m,k}\right\rangle }_{k=1}^{K}. \end{array}$$ (21)

Afterwards, the source-level scores are combined by means of a pooling function (Eq (22)), such as an average, obtaining mixture-level score sequences, $\hat{D}=\;{\langle {\hat{d}}_{k}\rangle }_{k=1}^{K}$.

$$\begin{array}{c} f^{\left(pool\right)}\left({\left\langle {\hat{d}}_{m,k}\right\rangle }_{m=1}^{M}\right)={\hat{d}}_{k}, \end{array}$$ (22)

In order to train the JSS model, we propose an iterative process. First, the Source Separation and the Sound Event Detection blocks are pre-trained separately. The SED block is pre-trained in the same manner as the DCASE SED Baseline system, using Mean Teacher semi-supervised training, whereas two different pre-training methods are considered for the SSep block: a supervised pre-training with PIT (Eq (12)), and an unsupervised pre-training with MixIT (Eq (17)).

**Fig 6:**
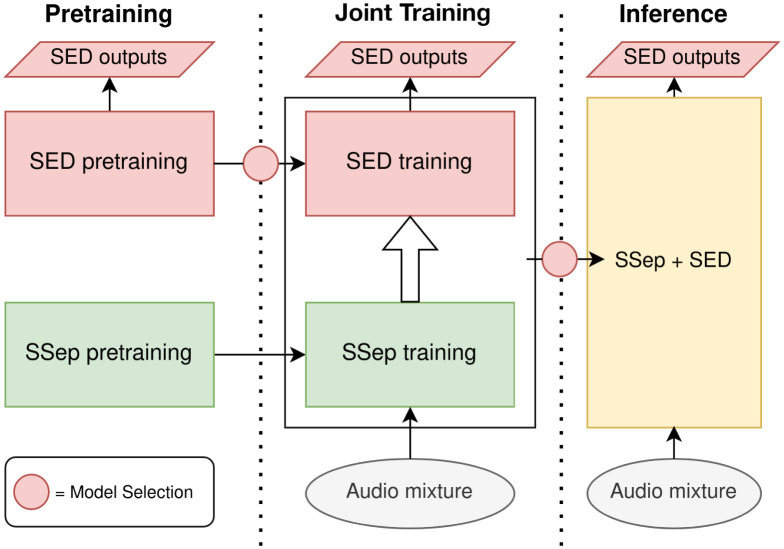
Block diagram of Joint training for Joint Source Separation + Sound Event Detection.

**Fig 7:**
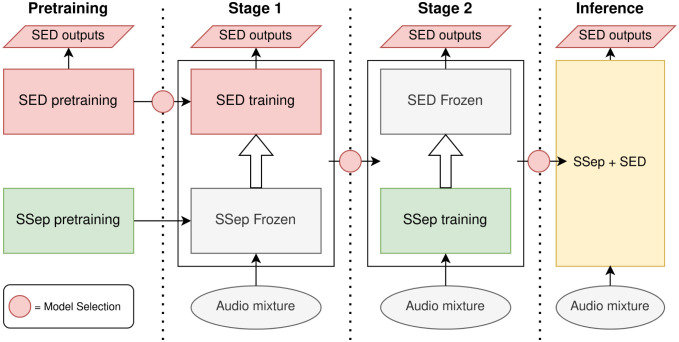
Block diagram of Two-stage training for Joint Source Separation + Sound Event Detection.

Afterwards, taking the pre-trained blocks as a starting point, we compare two training methods. On the one hand, Joint Training (JT) performs a single training process, updating the weights of both blocks (***θ***_sep_ and ***θ***_sed_) simultaneously (Fig 6). Alternatively, Two-stage Training (TST) performs two additional training processes (Fig 7), the first one updating only ***θ***_sed_ (Stage 1), and the second one updating only ***θ***_sep_ (Stage 2).

The main motivation for training the SSep and SED blocks together is that the separation artifacts introduced by the SSep block in audio signals create a domain mismatch with the pre-trained SED block, requiring a fine-tuning to separated sources.

The purpose of Two-stage Training, in contrast to Joint Training, is to control the convergence of each block independently. The first stage aims to adapt the SED block to the separated sources estimated by the SSep block, solving the domain mismatch without updating ***θ***_sep_. Afterwards, Stage 2 is approached as a fine-tuning of the SSep separation block to the SED task. Additionally, each stage can provide further insights on the impact of each block to the final performance.

In both JT and TST, the Mean Teacher method is applied, in order to deal with different levels of annotation in training data. The loss function employed is the SED objective *L*_sed_, described in Eq (6).

### Mean Teacher model selection

The aim of the training process is to find the optimal set of weights, θ*, that maximizes the performance in external data. For this purpose, the model is tested over a validation data set after each training epoch, computing an objective metric *P*_*obj*_. When the training is finished, the set of weights that maximizes *P*_*obj*_ is selected as the best model.

Moreover, the Mean Teacher training process updates two models in parallel: student and teacher. Therefore, at each training epoch *i*, the training has learnt two different sets of weights, $\theta_{i}^{\text{(s)}}$, for the student, and $\theta_{i}^{\text{(t)}}$, for the teacher. As described in Eq (3), the weights of the teacher are obtained as an exponential moving average of the student weights in previous steps, resulting in a smoother version of the student.

The DCASE SED Baseline model selection finds the best epoch, *b*, according only to the student models (Eq (23)). Then, at test time, the student and teacher models at epoch *b*, with weights $\theta_{b}^{\text{(s)}}$ and $\theta_{b}^{\text{(t)}}$ respectively, are evaluated over the dev-test data in terms of PSDS and *F*_1_ scores. The decision whether to choose the student or the teacher model for external data (e.g. the DCASE evaluation dataset) can be made at this point, but it is usually observed that the teacher model gives better results.

$$\begin{array}{c} b=\text{arg}\;\underset{i}{\text{max}}\;P_{obj}\left(\theta_{i}^{\text{(s)}}\right). \end{array}$$ (23)

The proposed training methods for JSS, especially Two-stage Training, require several Mean Teacher training processes, each of them involving a model selection decision (Fig 7). Therefore, model selection is particularly relevant in JSS.

In order to enhance the model selection criterion employed by the baseline, we propose a teacher model selection, which searches the best epoch *b*^(t)^ considering the teacher models at each training epoch *i* (Eq (24)). In this manner, we select the best teacher model, with weights $\theta_{b^{\text{(t)}}}^{\text{(t)}}$, instead of the best student model, with weights $\theta_{b}^{\text{(s)}}$, according to *P*_*obj*_.

$$\begin{array}{c} b^{\text{(t)}}=\text{arg}\;\underset{i}{\text{max}}\;P_{obj}\left(\theta_{i}^{\text{(t)}}\right). \end{array}$$ (24)

Considering that teacher models often perform better than students at test time, even when selecting the best epoch with student models, the proposed criterion is expected to provide enhanced results. Moreover, the adequacy of model weight averaging for generalization in deep neural networks has already been discussed in other training techniques, such as Stochastic Weight Averaging [32], supporting the hypothesis that teacher models, constructed by averaging the weights of students (Eq (3)), provide enhanced robustness.

## Experimental framework

### Proposed experiments

Our experimental settings aim to compare the Sound Event Detection performance of the JSS methods (Joint Training and Two-stage Training) with two baseline systems, both proposed by the DCASE Challenge: a SED system and a SSep+SED system.

Moreover, the experiments are designed to offer a comparison of the different methods described for JSS:

- **Joint Training vs. Two-stage Training.** Whereas JT requires less training processes, TST ensures an independent convergence for the SED and the SSep blocks, training each one of them in different stages.
- **Supervised vs. Unsupervised Source Separation pre-training.** The supervised pre-training for SSep requires a SSep dataset with oracle sources available, limiting the use of in-domain data. On the other hand, unsupervised pre-training with MixIT allows the use of in-domain data, even without reference sources available.
- **Student model selection vs. Teacher model selection.** In contrast with the default model selection criterion for Mean Teacher in the DCASE Baseline, our proposed model selection aims to provide enhanced robustness by taking into account the performance of the teacher models.

In order to compare the aforementioned settings, the two PSDS scenarios proposed by the DCASE Challenge are considered as performance metrics, as well as the event-based *F*_1_ score. In addition to global performance, class-wise metrics are provided, so the goodness of each system for specific event categories can be compared.

Moreover, aiming to compare the proposed JSS models, which are composed of a single branch (Fig 5), with the SSep-SED Baseline of DCASE, which is a combination of two separate branches (Fig 3), we evaluate different model combinations that are computed as late fusions, averaging the mixture-level SED score sequences ($\hat{D}$). The combination procedure for *N* models is described in Eq (25).

$$\begin{array}{c} {\hat{D}}^{\left(comb\right)}=\frac{1}{N}\sum_{n=1}^{N}{\hat{D}}^{\left(n\right)}. \end{array}$$ (25)

### Datasets

#### DESED: Domestic Environment Sound Event Detection

DESED [9, 10] is the Sound Event Detection dataset for DCASE Task 4, and it is composed of 10-second audio clips with different origins and types of annotations. According to the source of the audio, the available labels, and the purpose of the data, several subsets are defined:

- **Weak training:** 1578 recordings obtained from AudioSet, including the clip-level annotations for target events.
- **Unlabeled training:** 14412 clips obtained from AudioSet, with no annotations available.
- **Synthetic training:** 12500 artificial audio mixtures created by overlapping recordings of foreground events (obtained from FSD) and background sounds (from the SINS dataset [33]). The Scaper toolkit [34] is used to generate the mixtures, also providing their strong annotations.
- **Validation:** 1168 clips obtained from AudioSet, and manually annotated with strong labels.
- **Public evaluation:** 692 clips obtained from AudioSet, and manually annotated with strong labels.

The first three subsets (Weak, Unlabeled, and Synthetic) are intended for model training, whereas the Validation subset serves as development data during the challenge in order for participants to choose their best systems. The Public evaluation subset is an additional test dataset, which aims to assess whether the decisions made over the Validation set generalize to different data.

In addition to the described subsets of DESED, we consider a complementary test dataset: the Public overlap set, proposed in one of our previous analyses of the Sound Event Detection task [20], is designed to evaluate the performance of SED systems in conditions of severe overlap between different events, which have been shown to represent a challenging scenario for accurate event detection. In order to obtain the audio mixtures of the Public overlap set, the audio segments of the DESED Public evaluation set are randomly added together in pairs, joining their ground truth annotations accordingly. The Public overlap set is formed by three permutations of the Public evaluation segments, resulting in 2076 audio mixtures (three times the size of the Public evaluation set).

It should be noted that the Public overlap set is designed to represent artificial co-occurrence of sound events, which could have a different impact on the performance of the models compared to naturally overlapped sounds (i.e., several sound sources being recorded at the same time). Although natural overlap can be found in other DESED datasets, an analysis of performance under such kind of overlap would present certain limitations, due to the lack of annotations for non-target events and the scarcity of examples of overlap between target events [35].

#### FUSS: Free Universal Source Separation

FUSS [30] is a Universal Source Separation dataset, built by means of audio overlapping. In order to obtain the artificial mixtures contained in FUSS, a background recording and one to three foreground recordings with different sound categories are summed together, in a similar way to DESED synthetic audios. The background and foreground recordings are obtained from FSD.

The dataset contains 20000 training mixtures, 1000 mixtures for validation (i.e. model selection) and 1000 for test. Given that all of them are artificially composed, the individual sources are available as training targets, allowing the use of supervised algorithms for Source Separation training.

#### YFCC100M: Yahoo-Flickr Creative Commons

YFCC100M [36] is a large-scale multimedia dataset formed by free-licensed videos and pictures obtained from web sources. Although individual sources of the audio from the nearly 800000 videos are not provided, the audio tracks can be used as Universal Source Separation training data by means of an unsupervised approach such as MixIT [13].

### Model settings

#### Sound Event Detection baseline settings

Regarding the SED block, our models share their structure and settings with the SED Baseline of DCASE 2021 Task 4. Such model is a CRNN with a convolutional stage of 7 layers and a recurrent stage of 2 Bidirectional Gated Recurrent Units (Bi-GRU) [23], implemented with *pytorch* [37].

Mean Teacher training is employed, with a learning rate of 10^−3^ and an EMA factor of *α*_ema_ = 0.999. Mixup data augmentation [38] and dropout regularization [39] are applied, with 0.5 probability each. Median filtering is applied to the output prediction scores with a filter length of 450ms.

The SED baseline is fed with mel-spectrogram features of the audio segments (sampled at 16kHz), with 128 mel filters, Hamming windows of *L* = 2048 samples, spaced by *R* = 256 samples, and Fast Fourier Transforms (FFT) of *N* = 2048 samples. This feature configuration is kept for the rest of the models.

In terms of data distribution, the training set is formed by the DESED Unlabeled, Weak and Synthetic data sets, from which 10% of the weak set and 20% of the synthetic set are reserved as validation data to perform model selection. The DESED Validation (dev-test) and Public Evaluation sets are used as test data.

In the SED Baseline, model selection is performed using the student model (Eq (23)). The objective metric $P_{\text{obj}}^{\text{(Bs)}}$ (Eq (26)) is the macro-averaged *F*_1_ score obtained by the student over the validation subset, formed by weak and synthetic training segments. An intersection-based *F*_1_ score is computed over synthetic data, whereas weak *F*_1_ is computed for the weakly-labeled data.

$$\begin{array}{c} P_{\text{obj}}^{\left(Bs\right)}=F_{1}^{\text{(int)}}(\theta^{\text{(s)}};X_{\text{synth}}^{\text{(val)}},Y_{\text{synth}}^{\text{(val)}})+F_{1}^{\text{(weak)}}(\theta^{\text{(s)}};X_{\text{weak}}^{\text{(val)}},Y_{\text{weak}}^{\text{(val)}}) \end{array}$$ (26)

#### SSep+SED baseline settings

The SSep+SED Baseline involves pre-trained models for SSep and SED. Whereas the SED stage of the system is identical to the SED Baseline, the SSep model is an Improved Time-Domain Convolutional Network (TDCN++) [13], similar to ConvTas-Net, with *M* = 8 outputs. The model, implemented in TensorFlow, is trained in an unsupervised fashion using MixIT, employing 1600 hours of audio from YFCC100M as training data.

The score pooling function used in this baseline is a sum, meaning that the mixture-level scores for class *k*, ${\hat{d}}_{k}$, are the sum of the predictions over the estimated sources, as described in Eq (27).

$$\begin{array}{c} f^{\left(pool\right)}\left({\left\langle {\hat{d}}_{m,k}\right\rangle }_{m=1}^{M}\right)=\sum_{m=1}^{M}{\hat{d}}_{m,k}={\hat{d}}_{k} \end{array}$$ (27)

In this system, Source Separation is performed as an offline process. Therefore, the pre-trained SSep model is not updated or fine-tuned to the SED task or the DCASE data. The fine-tuning process relies on the same configuration as the SED Baseline, including mel-spectrogram features, Mean Teacher training and model selection.

#### Joint Source Separation + Sound Event Detection settings

In contrast with the SSep+SED Baseline, our proposed methods use a ConvTas-Net model for Source Separation. This model is implemented in *pytorch* within the audio separation toolkit Asteroid [40], allowing for better integration with the SED Baseline. The structure of the ConvTas-Net is configured with *M* = 4 outputs, *R* = 1 repeat and *X* = 4 convolutional blocks.

The pre-training of the ConvTas-Net SSep block is performed either using PIT (supervised) or MixIT (unsupervised). In the case of supervised training, the FUSS dataset is used as training and validation data, whereas the unsupervised training with MixIT uses data from DESED. In particular, the DESED Synthetic and Unlabeled training sets are used for training, and the Weak training set is used for validation.

The pre-training of the SED stage in JSS is the same as in the Baseline systems. The mel-spectrogram and Mean Teacher training settings are preserved for the posterior stages of JSS, whereas the learning rate is decreased after each model selection: to 10^−4^ in Joint Training and Stage 1 of Two-stage Training, and to 10^−5^ in Stage 2.

In order to combine source-level scores into mixture-level scores, JSS models use a max-pooling function, defined in Eq (28).

$$\begin{array}{c} f^{\text{(pool)}}\left({\left\langle {\hat{d}}_{m,k}\right\rangle }_{m=1}^{M}\right)=\underset{m}{\text{max}}{\left\langle {\hat{d}}_{m,k}\right\rangle }_{m=1}^{M}={\hat{d}}_{k} \end{array}$$ (28)

Model selection is applied either with the default method (student model selection, Eq (23)) or with the proposed teacher model selection (Eq (24)). In the latter case, the default objective function is computed using the teacher models (***θ***^(t)^), as is shown in Eq (29). Then, at test time, the best teacher model is used for inference.

$$\begin{array}{c} P_{\text{obj}}^{\text{(t)}}=F_{1}^{\text{(int)}}(\theta^{\text{(t)}};X_{\text{synth}}^{\text{(val)}},Y_{\text{synth}}^{\text{(val)}})+F_{1}^{\text{(weak)}}(\theta^{\text{(t)}};X_{\text{weak}}^{\text{(val)}},Y_{\text{weak}}^{\text{(val)}}) \end{array}$$ (29)

## Results

### Results of individual models

The performance obtained by each of the proposed methods is measured in terms of PSDS and event-based *F*_1_ score. Following the evaluation rules of the DCASE Challenge, the scenarios PSDS1 and PSDS2 are considered, and the macro-averaged event-based *F*_1_ score is computed with a 200ms collar for onsets and a collar length of max(200*ms*, 0.2*l*) for offsets, where *l* is the length of the event.

Regarding JSS, results are provided for the Joint Training (JT) and Two-stage Training (TST) methods, dividing TST into Stage 1 (S1) and Stage 2 (S2). The results of the initial state of the JSS model (a concatenation of the pre-trained blocks for SSep and SED) are included as Stage 0 (S0). The performances of the SED and the SSep+SED Baselines (SED Bs and SSep-SED Bs) are provided as benchmarks.

Table 2 shows the global performance of the SED Baseline and the proposed models over the DESED Validation and DESED Public evaluation sets, in terms of the three considered metrics. Since the DCASE SSep-SED baseline is in fact a combination of a SSep+SED system and a SED system (Fig 3), its results will be included next to the model combinations.

**Table 2:** Sound Event Detection results obtained with the DCASE 2021 SED Baseline system (SED Bs) and the Joint Source Separation + Sound Event Detection proposed methods: Pre-trained model (S0), Two-stage Training (S1, S2), and Joint Training (JT). Results are provided over the DESED Validation (dev-test) and Public evaluation sets, in terms of PSDS1, PSDS2 and event-based *F*_1_ score. Two pre-training methods for Source Separation (FUSS and DESED) and two model selection criteria (Student and Teacher) are compared. The best results for each metric/dataset are highlighted in bold.

	Validation (dev-test)						Public evaluation					
	Student model sel.			Teacher model sel.			Student model sel.			Teacher model sel.		
	PSDS1	PSDS2	F1(%)	PSDS1	PSDS2	F1(%)	PSDS1	PSDS2	F1(%)	PSDS1	PSDS2	F1(%)
SED Bs	0.338	0.522	40.12	0.357	0.552	41.65	0.372	0.582	43.89	0.398	0.613	44.96
FUSS-S0	0.241	0.336	31.42	0.281	0.380	33.15	0.280	0.367	36.60	0.305	0.410	39.90
FUSS-S1	0.329	0.517	41.03	0.349	0.534	41.47	0.364	0.562	41.15	0.393	0.587	44.49
FUSS-S2	0.344	0.549	42.36	0.356	0.552	42.75	0.376	0.587	44.14	0.399	0.615	44.14
FUSS-JT	0.336	0.535	41.50	0.358	0.547	41.46	0.361	0.576	44.54	0.402	0.592	44.85
DESED-S0	0.249	0.352	33.89	0.273	0.373	35.69	0.302	0.396	38.03	0.327	0.383	39.37
DESED-S1	0.346	0.539	39.25	0.355	0.550	42.41	0.374	0.569	42.60	0.390	0.613	45.89
DESED-S2	0.328	0.529	40.85	0.362	**0.572**	**43.40**	0.392	0.594	43.60	0.391	**0.638**	43.41
DESED-JT	0.337	0.504	40.77	**0.365**	0.555	43.14	0.394	0.573	**46.05**	**0.409**	0.602	45.10

Most conclusions are similar for both of the datasets. Generally, the proposed teacher model selection provides better results than the default model selection, which is based on the student models. Although the change in the model selection strategy was motivated by the existence of several model selection decisions in the proposed methods, this improvement is observed also in the baseline system. Therefore, it is shown that the teacher model selection method is also beneficial for regular Sound Event Detection models trained with Mean Teacher. Regarding the two proposed pre-training methods for the Source Separation block, better performance is usually obtained when employing Mixture Invariant Training over the DESED dataset, underlining the ability of unsupervised source separation to leverage in-domain data when the individual target sources are not available.

When comparing the performance of Two-Stage Training and Joint Training (considering teacher model selection), the results show that Joint Training is slightly better at PSDS1, whereas Two-Stage Training (at Stage 2) provides higher PSDS2, achieving in both cases slightly better performance than the SED baseline. The results of the different stages of TST give some insights about their impact in the final performance: First, the results of the initial state of the joint model, Stage 0, are considerably worse than the Baseline, indicating that the pre-trained SSep block introduces a domain mismatch with respect to the original mixtures. The first stage (S1), which fine-tunes the SED block, lowers this gap in performance, yielding results closer to the Baseline. Stage 2 is able to improve the results of S1 by fine-tuning the SSep block for the Sound Event Detection task.

### Results of combined models

Aiming to allow a comparison with the SSep-SED Baseline of DCASE 2021, which is itself a combination of a SSep+SED system and a SED system, several model combinations are defined between the SED Baseline and the JSS models (with DESED SSep pre-training and teacher model selection). Their results are gathered in Table 3, showing that all the combinations outperform the SSep-SED baseline in terms of PSDS1 and PSDS2 over the Validation set. However, only the combinations which include both DESED-S2 and DESED-JT models are able to obtain a higher *F*_1_ score than the SSep-SED baseline system. When observing the results over the Validation set, most of the fusions obtain lower PSDS1 than the SSep-SED baseline, and none of them is able to obtain a higher *F*_1_ score. Nonetheless, an improvement in PSDS2 is obtained by the combinations that include DESED-S1 or DESED-S2.

**Table 3:** Sound Event Detection results obtained with the DCASE 2021 SSep+SED Baseline system (SSep-SED Bs) and JSS model fusions over the DESED Validation (dev-test) and Public evaluation sets, in terms of PSDS1, PSDS2, and event-based F1 score. All the models in each fusion use Teacher model selection. The best results for each metric/dataset are highlighted in bold.

	Validation (dev-test)			Public evaluation		
	PSDS1	PSDS2	F1(%)	PSDS1	PSDS2	F1(%)
SSep-SED Bs	0.363	0.532	44.34	0.424	0.616	**48.32**
SED Bs + DESED-S1	0.381	0.585	44.07	0.420	0.648	46.85
SED Bs + DESED-S2	0.379	**0.590**	43.74	0.415	0.655	46.67
SED Bs + DESED-JT	0.366	0.563	43.02	0.413	0.612	45.56
SED Bs + DESED(S2+JT)	0.379	0.587	45.05	0.421	0.652	46.79
SED Bs + DESED(S1+S2+JT)	**0.384**	**0.590**	44.81	**0.425**	**0.660**	46.06
DESED(S2+JT)	0.380	0.589	**45.52**	0.421	0.654	46.00
DESED(S1+S2+JT)	0.381	0.587	45.13	0.421	0.655	45.24

### Results under event overlap

Source Separation as an auxiliary task for Sound Event Detection should be especially helpful in situations when several events coincide in the same lapse of time. For that reason, we assess the performance of the JSS models over the Public overlap set [20], providing the results in Table 4.

**Table 4:** Sound Event Detection results obtained with the DCASE 2021 SED Baseline system (SED Bs) and the Joint Source Separation + Sound Event Detection proposed methods: Initial model (S0), Two-stage Training (S1, S2), and Joint Training (JT). Results are provided over the DESED Public overlap set [20], in terms of PSDS1, PSDS2 and event-based *F*_1_ score. Two pre-training methods for Source Separation (FUSS and DESED) and two model selection criteria (Student and Teacher) are compared. The best results for each metric are highlighted in bold.

	Public overlap					
	Student model sel.			Teacher model sel.		
	PSDS1	PSDS2	F1(%)	PSDS1	PSDS2	F1(%)
SED Bs	0.144	0.319	23.18	0.174	0.352	25.16
FUSS-S0	0.096	0.187	23.50	0.123	0.221	25.02
FUSS-S1	0.168	0.336	23.97	0.184	0.352	24.96
FUSS-S2	0.159	0.341	23.84	0.176	0.354	24.27
FUSS-JT	0.149	0.320	23.93	0.171	0.341	24.95
DESED-S0	0.155	0.269	25.80	0.179	0.277	**27.70**
DESED-S1	0.172	0.341	25.59	**0.200**	**0.378**	27.39
DESED-S2	0.166	0.345	25.28	0.188	0.371	26.21
DESED-JT	0.171	0.332	26.76	0.187	0.355	26.00

The teacher model selection criterion and the unsupervised Source Separation pre-training with DESED are also beneficial for this scenario, however, the best results in PSDS 1 and 2 are obtained by the S1 models, and the best *F*_1_ result is held by the S0 model. These behaviors could be explained by considering that the generation process of the dataset (artificially overlapping sound mixtures) and the unsupervised pre-training of the Source Separation block (separating mixtures of mixtures) can be seen as opposite operations. In other words, the pre-trained SSep block is already prepared to deal with the same kind of data that is present in the Public overlap set, therefore the fine-tuning performed in Stage 2 or Joint Training does not provide any advantage in this scenario.

The results of the combined models over the Public overlap set are shown in Table 5. In this case, the SSep-SED Baseline yields the best PSDS1 result, while every combination that includes S1 or S2 obtains better PSDS2 than the baseline. The *F*_1_ result of the baseline is only outperformed by the combinations that include the Stage 1 model. This results follow a similar behavior to those of the individual models.

**Table 5:** Sound Event Detection results obtained with the DCASE 2021 SSep+SED Baseline system (SSep-SED Bs) and JSS model fusions over the DESED Public overlap set, in terms of PSDS1, PSDS2, and event-based F1 score. All the models in each fusion use Teacher model selection. The best results for each metric are highlighted in bold.

	Public overlap		
	PSDS1	PSDS2	F1(%)
SSep-SED Bs	**0.208**	0.377	27.11
SED Bs + DESED-S1	0.204	0.395	27.45
SED Bs + DESED-S2	0.195	0.387	27.04
SED Bs + DESED-JT	0.185	0.363	25.80
SED Bs + DESED(S2+JT)	0.197	0.385	26.94
SED Bs + DESED(S1+S2+JT)	0.205	**0.397**	**27.59**
DESED(S2+JT)	0.199	0.383	27.27
DESED(S1+S2+JT)	0.206	0.394	27.43

## Further analysis and discussion

Although the results show that the proposed methods yield small but consistent improvements in the DCASE Sound Event Detection task, further analysis can provide a better understanding about the role and impact of Source Separation in SED.

For this purpose, we study three additional aspects: First, the class-wise results of the JSS models, which allow us to analyse the impact of Source Separation in different kinds of events. Then, given that the main motivation for Source Separation as a pre-processing step for SED is to isolate different audio events in separate sources, we propose a metric that aims to assess to what extent this is achieved in the different proposed models. Finally, we provide graphical representations for some test examples, including the sources estimated by the SSep block as well as the source-level and mixture-level SED scores.

### Class-wise results

**Fig 8:**
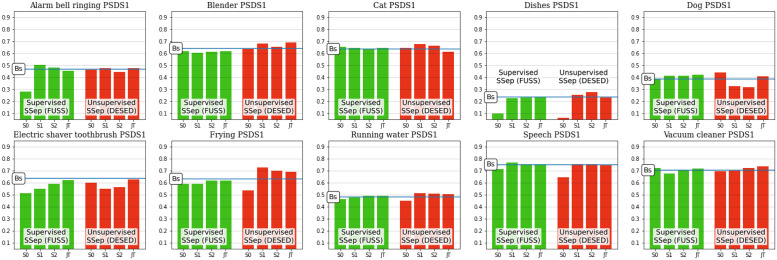
Class-wise PSDS1 results of individual models with teacher model selection over DESED Public evaluation data. The SED Baseline performance is indicated with a blue horizontal line.

**Fig 9:**
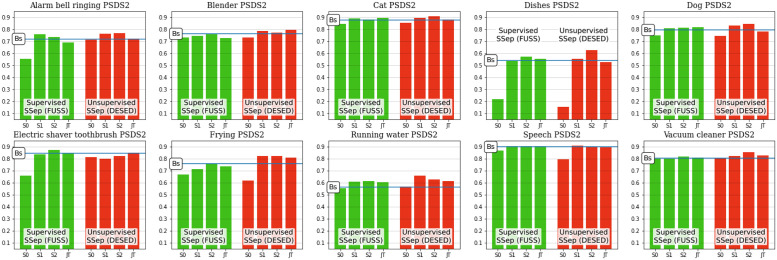
Class-wise PSDS2 results of individual models with teacher model selection over DESED Public evaluation data. The SED Baseline performance is indicated with a blue horizontal line.

The evaluation framework of DCASE Challenge Task 4 is mainly focused on the global performance of the models over the whole set of 10 event categories. However, the different target events are noticeably diverse in terms of acoustic characteristics, for instance, regarding their duration or their spectral properties [35], therefore some models could be more fitted to correctly detecting a certain subset of event categories [41]. This motivates a class-wise analysis of the results, aiming to better understand the behaviour of the JSS systems for different event classes. For this purpose, we provide the class-wise results of the proposed models (employing Teacher model selection) over the Public evaluation set in Figs 8 and 9.

The class-wise results suggest that the domain mismatch introduced by Source Separation, which severely impacts the performance of Stage 0 models, does not affect all classes. In fact, this initial state performs equally, or even slightly better than the SED baseline in some event categories (e.g. *Cat*, *Dog* in terms of PSDS1, *Vacuum cleaner* in terms of PSDS2). This indicates that the artifacts introduced by Source Separation do not alter the identification of these events. Nevertheless, the detection of other classes is noticeably degraded, especially *Dishes*. This is the event category for which the SED baseline yields its worst performance, meaning that its correct detection is particularly difficult. Thus, in this class the SED block is less robust to the domain mismatch introduced in Stage 0.

When comparing the two different approaches to SSep pre-training, some differences can be observed. For instance, in Stage 0 models, some classes obtain clearly lower results with FUSS supervised pre-training than with DESED unsupervised pre-training (e.g. *Alarm bell ringing, Electric shaver toothbrush*). In contrast, other categories (*Speech*, *Frying*) show the opposite case, evidencing that each SSep block harms the SED performance in different ways. However, these differences are not observed in the subsequent stages of JSS (Stage 1, Stage 2, or Joint Training), meaning that the fine-tuning of the SED block is able to overcome the mismatches introduced by either supervised or unsupervised SSep blocks. Actually, it can even revert the situation, like in the case of *Frying*.

### Diversity of predictions across sources

The motivation for the use of Source Separation as an auxiliary task for Sound Event Detection is the idea that automatically separated sources can be more adequate inputs for Sound Event Detection. This hypothesis can be assessed by measuring the SED performance of the systems, as done in the Results section. However, an analysis of the interaction between SSep and SED in the proposed models would be able to provide more specific insights.

For this purpose, we have studied the diversity of Sound Event Detection predictions across the *M* different sources estimated for each sound mixture. We consider the source-level score sequences, ${\hat{D}}_{m}={\langle {\hat{d}}_{m,k}\rangle }_{k=1}^{K},m\in \left[1,M\right]$, and quantify the similarities between every source pair by means of cosine scoring. The similarity between the predictions of a pair of sources, (*m*, *n*) ∈ [1, *M*], *m* ≠ *n*, is computed in Eq (30) as the average of their cosine similarity at each time step *t*.

$$\begin{array}{c} s_{cos}\left({\hat{D}}_{m},{\hat{D}}_{n}\right)=\frac{1}{T}\sum_{t=1}^{T}\frac{{\hat{D}}_{m}\left(t\right)\cdot {\hat{D}}_{n}\left(t\right)}{\left|\right|{\hat{D}}_{m}\left(t\right)||\cdot \left|\right|{\hat{D}}_{n}\left(t\right)\left|\right|}\in \left[0,1\right] \end{array}$$ (30)

The total similarity score of an audio segment is computed as the average similarity of the SED scores for every pair of estimated sources. Given that the SED scores are bound between 0 and 1, the similarity score is as well.

**Fig 10:**
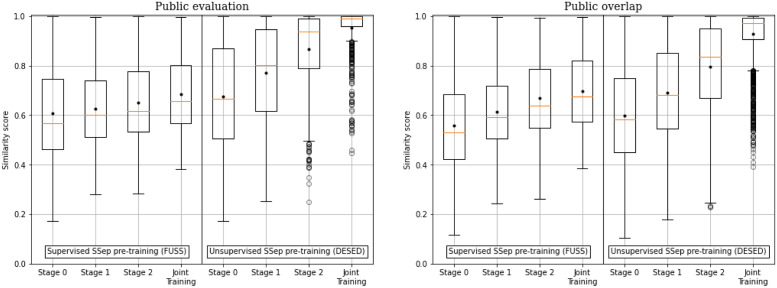
Distribution of the cosine similarities across source-level predictions of different models for DESED Public evaluation (left) and Public overlap (right).

The distribution of similarity scores for test segments using different JSS models is shown in Fig 10. In general, higher similarity scores are observed for Public eval data than for Public overlap data, which is expected, considering that a larger number of events in a mixture allows for a higher diversity of events in its estimated sources. An additional general observation is that supervised pre-training of the SSep block with FUSS results in less similar predictions than unsupervised pre-training with DESED.

Regarding the different models, the least similar predictions across sources are obtained by the initial state, Stage 0, and the similarity of predictions increases with each step of Two-stage training (stages 1 and 2). The most similar predictions, however, are obtained with Joint Training. This behavior is particularly noticeable in the systems with unsupervised pre-training of SSep, which could indicate that the unsupervised SSep block is more prone to forgetting source separation, to some extent, when fine-tuned for the SED task.

Overall, the JSS model with more similar predictions is DESED-JT, with most of the Public eval examples obtaining similarity scores higher than 0.9. In practice, this model is detecting almost the same events in every estimated source, meaning that Source Separation is not performing a relevant role.

**Fig 11:**
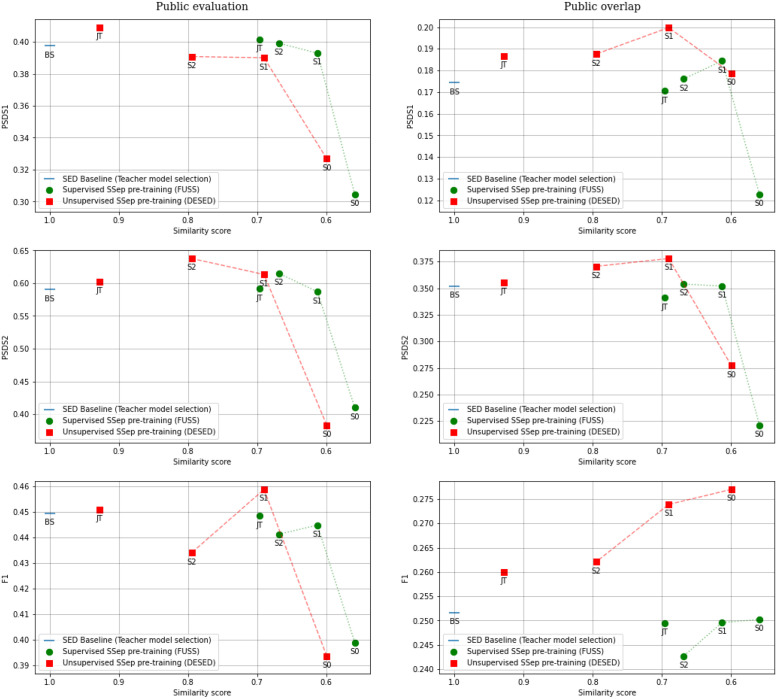
PSDS1, PSDS2 and F1 results of individual models with Teacher model selection over DESED Public evaluation (left) and Public overlap (right), plotted against their cosine-distance-based similarity scores. The SED Baseline is represented as a reference, considering that its similarity score is 1.

A comparison of mean similarity scores and SED performances of the different proposed models is provided in Fig 11. Although the similarity scores do not provide a complete explanation of the differences in performance, it can be observed that, with the exception of Stage 0 systems in some cases, more diverse predictions across estimated sources generally lead to better results in Public overlap data, whereas this does not happen in Public eval data, suggesting that effective Source Separation is more beneficial when tackling severely overlapped scenarios. This explains the lower performance of Joint Training or Stage 2 in overlapped data, when compared to Stage 1 (or even Stage 0, in terms of *F*_1_ score). However, this conclusion is limited due to the fact that the examples in Public overlap are artificially generated, which is an advantage for the SSep models employed (particularly for those pre-trained with DESED).

In conclusion, the proposed similarity score has allowed us to measure and observe the interactions between Source Separation and Sound Event Detection, highlighting the main difficulty of training SSep and SED systems jointly: when training JSS systems with a SED objective, the SSep block tends to forget its original task (decomposing the mixture into its different components), and provides instead similar signals for all its output channels. Although this issue does not necessarily harm SED performance (Fig 11), it does not align with the original motivation of JSS, which is to improve the detection performance by separating the input mixture into simpler components.

### Intermediate representations of Joint Source Separation and Sound Event Detection

So far, we have analysed the SED performance of the JSS models, both globally and class-wise, and we have observed the effect of Source Separation in the SED predictions by measuring the diversity of the SED scores across the estimated sources. In this section, we aim to complement the analysis of JSS, providing a global overview of the method by representing the inputs and outputs of each block: (1) The input mixture waveform and its mel-spectrogram. (2) The mel-spectrogram of each estimated source output by the SSep block. (3) The SED score sequences obtained for each estimated source. (4) The mixture-level SED score sequences, computed as a max-pooling across the source-level scores.

We illustrate the different stages of JSS with an example extracted from DESED Public evaluation. For this audio segment, we provide the representations of the models that employ unsupervised SSep pre-training (DESED) at the initial state (Stage 0), with Two stage Training (Stages 1 and 2), and with Joint Training.

**Fig 12:**
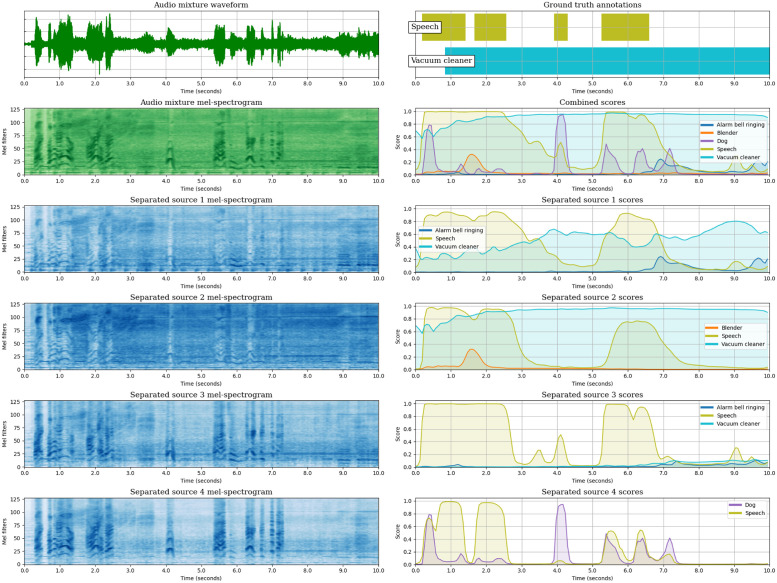
Visualization of the audio segment “AW9ZKFZKhDE_49_59”, from DESED Public evaluation, as processed by the JSS model DESED-S0 (Teacher model selection). The top left plot is the input mixture waveform, and below the mixture mel-spectrogram is shown. The four bottom mel-spectrograms in the left column are the sources estimated by the SSep block. Next to each source mel-spectrogram, its corresponding SED score sequences are represented. Over the source-level scores, the mixture-level score sequences are shown. Finally, the upper right plot represents the ground truth annotations of the segment. The figure is best viewed in color.

The example contains two target events mostly overlapped in time, *“Speech”* and *“Vacuum cleaner”*. The model at Stage 0 (Fig 12) correctly detects both events, but introduces false positive activations of other classes: *“Blender”*, *“Alarm bell ringing”* and, more noticeably, *“Dog”*, which illustrates the effect of the domain mismatch.

**Fig 13:**
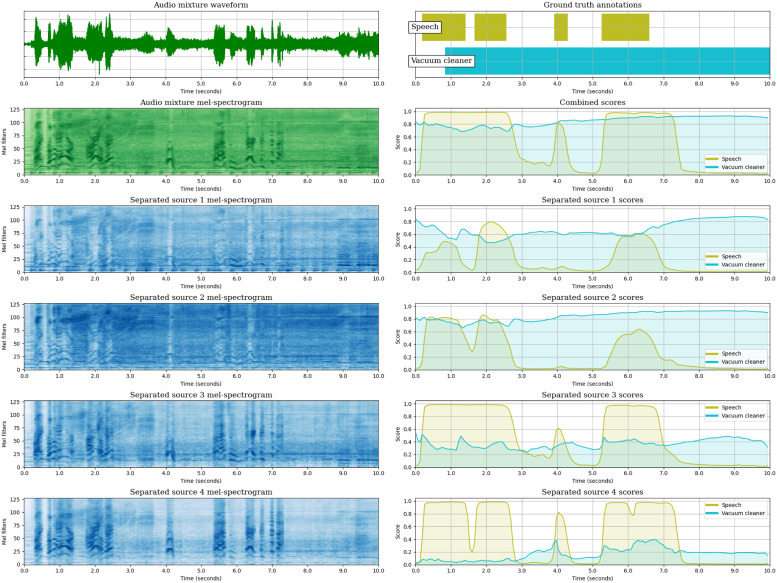
Visualization of the audio segment “AW9ZKFZKhDE_49_59”, from DESED Public evaluation, as processed by the JSS model DESED-S1 (Teacher model selection). The top left plot is the input mixture waveform, and below the mixture mel-spectrogram is shown. The four bottom mel-spectrograms in the left column are the sources estimated by the SSep block. Next to each source mel-spectrogram, its corresponding SED score sequences are represented. Over the source-level scores, the mixture-level score sequences are shown. Finally, the upper right plot represents the ground truth annotations of the segment. The figure is best viewed in color.

After the fine-tuning of the SED block in Stage 1 (Fig 13), these false positives are solved. In this stage, both events are detected in the four estimated sources, but the confidences in each one of them are different: the first two sources obtain high confidence for *“Vacuum cleaner”*, but a moderate confidence for *“Speech”*, even skipping one of its appearances. In contrast, a higher confidence for *“Speech”* is observed in the two last sources (which present cleaner speech mel-spectrograms), whereas the confidence for the detection of *“Vacuum cleaner”* is much lower. This suggests that the correct separation of events is helpful in order to enhance the confidence of the detections.

**Fig 14:**
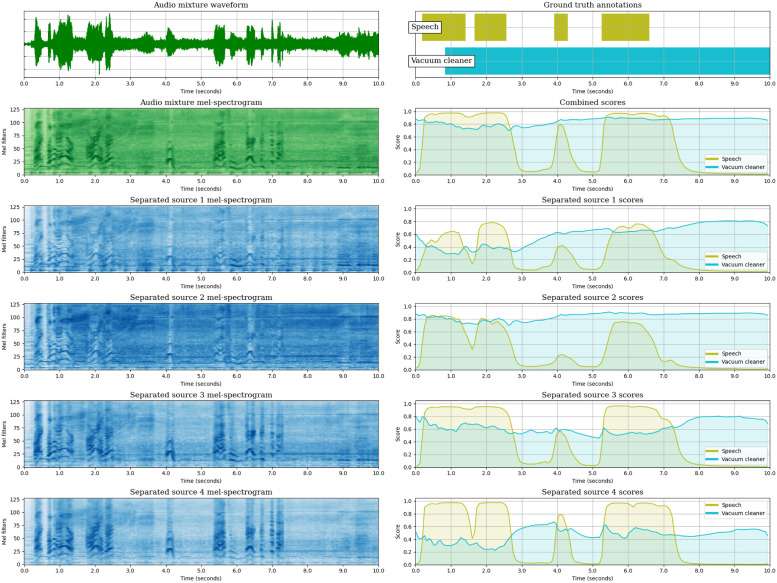
Visualization of the audio segment “AW9ZKFZKhDE_49_59”, from DESED Public evaluation, as processed by the JSS model DESED-S2 (Teacher model selection). The top left plot is the input mixture waveform, and below the mixture mel-spectrogram is shown. The four bottom mel-spectrograms in the left column are the sources estimated by the SSep block. Next to each source mel-spectrogram, its corresponding SED score sequences are represented. Over the source-level scores, the mixture-level score sequences are shown. Finally, the upper right plot represents the ground truth annotations of the segment. The figure is best viewed in color.

In Stage 2 (Fig 14), after the fine-tuning of the SSep block, the final scores are very similar to those of Stage 1. However, the source-level scores have become less diverse than in Stage 1, with higher confidence for *“Vacuum cleaner”* in the last two sources. This suggests that the SSep fine-tuning decreases the ability of the SSep block to isolate different events into different sources.

**Fig 15:**
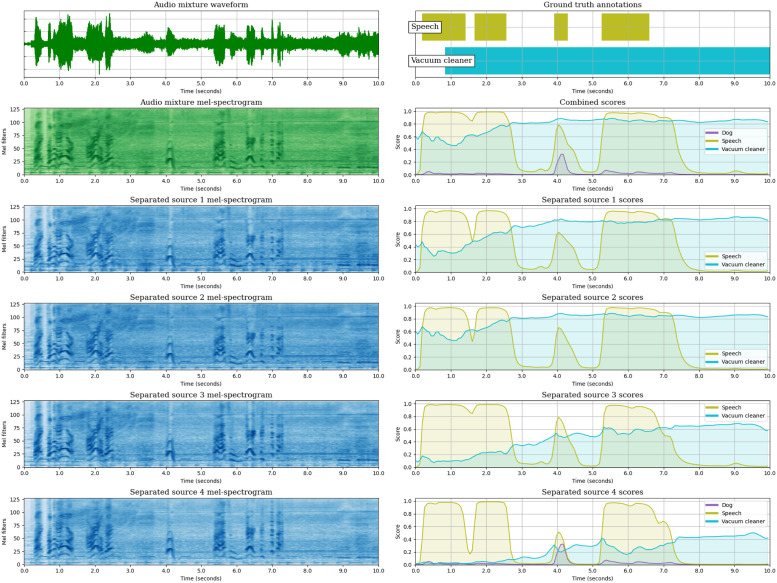
Visualization of the audio segment “AW9ZKFZKhDE_49_59”, from DESED Public evaluation, as processed by the JSS model DESED-JT (Teacher model selection). The top left plot is the input mixture waveform, and below the mixture mel-spectrogram is shown. The four bottom mel-spectrograms in the left column are the sources estimated by the SSep block. Next to each source mel-spectrogram, its corresponding SED score sequences are represented. Over the source-level scores, the mixture-level score sequences are shown. Finally, the upper right plot represents the ground truth annotations of the segment. The figure is best viewed in color.

Finally, in the case of Joint Training (Fig 15), the four estimated sources are very similar to the mixture, indicating that the joint fine-tuning of SSep and SED is also detrimental for the separation properties of the SSep block. In consequence, the source-level detection scores are mostly redundant. Nevertheless, the predictions for the example are generally correct, except for a false positive detection of *Dog*, which was present at Stage 0.

## Conclusions and future work

In this work, we define and analyze a method for Sound Event Detection that includes Source Separation as an integral component of the neural network structure. With respect to other related works in the field, the proposed Joint Source Separation and Sound Event Detection (JSS) method allows an explicit interaction between SSep and SED, thanks to a joint model that is trained in an end-to-end fashion, built from two pre-trained neural networks, for SSep and SED respectively.

The experimental framework is configured according to the DCASE Challenge Task 4, “Sound Event Detection and Separation in Domestic Environments”, providing results over the DESED Validation and Public evaluation sets. Moreover, given that the benefits of the SSep stage should be more evident when dealing with highly overlapped data, we offer results over an additional dataset containing sound mixtures severely affected by event overlap. In all of the studied data sets, the proposed models outperform the benchmark set by the DCASE SED Mean Teacher baseline in terms of two different PSDS scenarios.

Considering that SSep pre-training is required for JSS, the availability of in-domain SSep training data could become a limiting factor for the use of JSS. However, our experiments show that unsupervised SSep with Mixture Invariant Training is an adequate choice for the SSep pre-training step, even reaching better performance than supervised SSep pre-training over out-of-domain data. Additionally, our proposed model selection strategy for Mean Teacher, based on the teacher models, provides consistent improvements in SED performance at test time: such strategy is suitable not only for JSS models, but also for other mean-teacher-based SED models and, possibly, for Mean Teacher training in different applications.

Finally, aiming to provide further analysis and discussion of the role of Source Separation in the proposed models, we propose a study on the diversity of event predictions across different separated sources. The results have helped to highlight the main limitation of the proposed joint training methods: when training the Source Separation stage jointly with the Sound Event Detection stage using a SED objective, the SSep stage tends to forget separating sources and provide the same (or very similar) output for all the sources. In future work, we consider that the joint training process should be enhanced to deal with this limitation, for instance, combining the SED loss function with a Source Separation loss term in order to encourage separation and detection in a multi-task learning fashion.
